# In Vitro Probiotic Modulation of Specific Dietary Complex Sugar Consumption in Fecal Cultures in Infants

**DOI:** 10.3390/microorganisms13102352

**Published:** 2025-10-14

**Authors:** Daniela Mollova, Vesselin Baev, Ilia Iliev

**Affiliations:** 1 Department of Biochemistry and Microbiology, Faculty of Biology, University of Plovdiv, Tzar Assen 24, 4000 Plovdiv, Bulgaria; iliailiev@uni-plovdiv.bg; 2 Department of Molecular Biology, Faculty of Biology, University of Plovdiv, Tzar Assen 24, 4000 Plovdiv, Bulgaria; baev@uni-plovdiv.bg

**Keywords:** infant microbiome, co-cultivation, prebiotic, modulation

## Abstract

Establishing the relative stability of the gastrointestinal microbiome after birth is a long and complex process, and it occurs under various influences. The human gut microbiome plays a crucial role in influencing an individual’s health and well-being across all stages of life. Breastfeeding, the introduction of solid food at a certain stage after birth, and the type of food largely determine the composition of the developing microbiome. The influence of probiotics on the early development of the microbiome is gaining increasing interest. The method of in vitro co-cultivation with probiotic strains provides a clearer picture of the influence of these microorganisms on the community and the functional changes that the infant’s microbiome undergoes. We used fecal samples to study this influence by conducting metagenomic sequencing to determine the composition of the microbiome and a series of cultivations to determine the absorption of various fibers and prebiotic sugars from breast milk. We found statistically significant differences in the absorption of prebiotic sugars isolated from breast milk, as well as better absorption of several substrates in the presence of a probiotic strain.

## 1. Introduction

The infant gut microbiome refers to the community of microorganisms that inhabit the digestive tract of newborns and infants. This microbial ecosystem comprises various microorganisms, including bacteria, viruses, fungi, parasites, archaea, and other microorganisms. Bacteria are the most prevalent and diverse group within the gut microbiome [1]. The human gut microbiome plays a crucial role in influencing an individual’s health and well-being across all stages of life [2]. The establishment of a stable gut microbiota in infants occurs in two key stages [3]. The first transition occurs shortly after birth, during the breastfeeding phase, when *Bifidobacterium* dominates. At this stage, the gut microbiota is highly variable and less resistant to changes compared to that of adults. The second transition takes place during weaning when solid foods are introduced alongside milk consumption. This phase fosters the development of a more complex, adult-like microbiome, primarily composed of *Bacteroidetes* and *Firmicutes* [4]. After this stage, the infant’s gut microbiota becomes more diverse, stable, and mature [5]. The development of the gut microbiota in early life is strongly shaped by infant feeding methods, such as breastfeeding and formula feeding. The choice of nutrition plays a crucial role in determining the composition and function of the infant gut microbiota, primarily due to variations in nutrient content, especially the presence of Human Milk Oligosaccharides (HMOs) [6].

### 1.1. Influence of Breastfeeding and Formula Feeding on the Development of the Infant Gut Microbiome

The crucial role of breastfeeding in shaping a healthy infant’s gut microbiome has gained increasing recognition in recent years. A key component of breast milk is HMOs. In the colon, HMOs are fermented mainly by *Bifidobacteria*, leading to the production of short-chain fatty acids (SCFAs), which help suppress the growth of opportunistic pathogens from the *Clostridiaceae*, *Enterobacteriaceae*, and *Staphylococcaceae* families [7]. 

Human breast milk contains a variety of microbiota, including both Gram-positive bacterial strains associated with the skin and those unrelated to it [8].

Formula-fed (FF) infants exhibit a more diverse gut microbiome than their breastfed counterparts. They tend to have a higher abundance of *Clostridiales* and *Proteobacteria* in their gut. Additionally, their microbiota contains greater concentrations of *Atopobium* and *Bacteroides* while having lower levels of *Bifidobacteria* compared to breastfed infants [9]. Formula feeding is also associated with a reduction in the overall number of gut bacteria, while simultaneously increasing microbial diversity. These differences in microbiota composition are largely due to the absence of HMOs and the higher protein content in formula milk. As a result, they promote the growth of a wider range of bacterial species, leading to a microbiota composition that differs significantly from that of breastfed infants [9].

### 1.2. Prebiotic and Probiotic Supplementation

Several studies have investigated the effects of adding prebiotics to infant formula on gut microbiome composition. [10]. Research indicates that enriching formula with HMOs such as 2′-fucosyllactose(2-FL) and lacto-N-neotetraose offers significant benefits [11]. These supplements not only support healthy growth but also encourage the proliferation of beneficial *Bifidobacteria*, aligning the gut microbiome more closely with that of breastfed infants. Additionally, the study indicated reduced bifidogenic activity when the formula was combined with breastmilk, suggesting a potential interaction between their components [10].

The most commonly studied and utilized probiotic species belong to the *Lactobacillus*, *Bifidobacterium*, and *Saccharomyces* genera [12]. Researchers are also investigating the use of bacteria derived from breast milk to develop infant formulas that more closely mimic the nutritional composition of natural breast milk. The addition of *Bifidobacterium* species and/or lactic acid bacteria, such as *Lactobacillus* strains, to infant formula is considered safe and widely accepted, with the potential to enhance immune responses. However, studies examining the effects of probiotic supplementation in infant formulas have not found a strong link between fecal *Bifidobacterium* levels and *Bifidobacterium* supplementation [13]. A systematic review of 12 randomized controlled trials further supports this finding, concluding that probiotic supplementation does not significantly increase *Bifidobacteria* or *Lactobacilli* counts nor reduce pathogen levels such as *Bacteroides* and *E. coli* [14]. Regardless of the specific probiotic strain, infants given probiotics had higher fecal levels of Lactobacillus. Another study showed that healthy infants who consumed formula enriched with *Lactobacillus rhamnosus* GG (LGG) had a higher rate of *Lactobacilli* colonization than those fed standard formula [15]. Additionally, in very low-birth-weight infants, supplementation with *Bifidobacterium breve* Bb12 promoted gut colonization by this bacterium and enhanced *Lactobacilli* growth compared to those who did not receive probiotics [16].

### 1.3. Introduction of Complementary Foods 

By the sixth month, breast milk alone can no longer provide sufficient nutrients and energy to meet the infant’s increasing metabolic needs. Therefore, introducing complementary foods becomes essential for proper physical growth and neurological development [17]. The transition from solely milk feeding to incorporating family foods into an infant’s diet leads to significant alterations in gut microbiota. During this phase, alpha diversity increases, and the microbial community shifts from being dominated by *Bifidobacterium* to being primarily composed of *Bacteroidetes* and *Firmicutes* [18]. 

While breastfeeding and formula feeding have been thoroughly examined, there is still a shortage of long-term studies investigating how fibers from solid foods influence the development of the infant gut microbiome. Dietary fiber is a carbohydrate in plant foods, such as whole grains, vegetables, fruit, and legumes, which have been dominant in human diets for millions of years [19]. Over the course of a million years, the human gut microbiota has played a crucial role in nutrition by breaking down lactose and cellulose, detoxifying harmful substances, and producing vitamins, signaling molecules, and other important compounds [20]. During the weaning stage—around 4 to 6 months—the microbiome undergoes significant diversification as the infant’s diet transitions from breast milk oligosaccharides to plant-based fibers [21]. This dietary change favors the growth of bacterial groups equipped with the specific carbohydrate-active enzymes needed to break down new glycans, setting the stage for an adult-like gut microbiome by approximately 3 to 5 years of age. Consequently, any disruptions to the nutritional sources available to these bacteria during this critical period may have far-reaching health consequences and could lead to a generational decline in microbial diversity, increasing the risk of allergies and chronic diseases [22]. 

The effects of probiotic strains on the uptake of different substrates by microbiomes are currently poorly studied. We attempted to shed more light on the cross-feeding process in the cocultivation of microbiomes.

Our study aimed to determine in vitro how probiotic strains can influence the absorption of various fiber food extracts and important oligosaccharides present in breast milk. How and to what extent does the composition of the microbiome in infants differ functionally from the microbiome in adults in terms of the absorption of different substrates? We tried to determine exactly which substrates from different microbiomes the specific probiotic strain had a significant impact on.

## 2. Materials and Methods

### 2.1. Strain and Fecal Samples

Fecal samples were obtained from two vaginally delivered full-term healthy children at 12 and 10 months of age and from one adult at 35 years. The infants were exclusively fed with breastmilk (BF) until the sixth month after birth. After the sixth month, they were normally fed solid food. One of the two babies named IF1 received an additional probiotic containing *Bifidobacterium animalis* subsp. *lactis* (BB-12, Jamieson, Baby probiotic drops, Toronto, ON, Canada) for a month before the sample was taken. All infants were discharged from the Hospital at 2–3 days of life. The study was conducted in accordance with the Declaration of Helsinki and approved by the Ethics Committee of the University of Plovdiv “Paisii Hilendarski” (Approval No. 8/02.04.2025) for studies involving humans. Informed written consent was obtained from each infant’s parents and adults. Infants’ stool samples were collected at home over one or two weeks by scraping feces from nappies immediately after defecation using a sterile spatula. The samples were then frozen in sterile tubes at −20 °C and transported to the laboratory within a week. In an anaerobic chamber, a 1/5 (*w*/*v*) dilution was prepared using at least 20 g of fecal matter, obtained by combining consecutive samples from the same infant. This mixture was suspended in a pre-reduced PBS solution containing 25% (*v*/*v*) glycerol, vortexed for 10 min, and stored in 20–30 mL aliquots at −80 °C until needed.

### 2.2. DNA Extraction, Sequencing, and Data Analysis

Genomic DNA was extracted using the QIAamp Fast DNA Stool Mini Kit (Qiagen, Düsseldorf, Germany) following the manufacturer’s instructions, with some modifications. Specifically, a pellet from 1 mL of fecal culture was washed with PBS (VWR Chemicals, Solon, OH, USA) and centrifuged at maximum speed at 4 °C. Cell lysis was carried out at 95 °C for 20 min. DNA was eluted in 50 µL of buffer ATE, and its concentration was measured using a Nanodrop Spectrophotometer (ND-1000; V3.8.1 program, Waltham, MA, USA). The V3–V4 hypervariable region of the 16S rRNA gene was amplified and sequenced using the NovaSeq Illumina platform with a 2 × 250 bp paired-end (PE) read at Novogene (Novogene Europe, Cambridge, UK). The amplicon size distribution was qualitatively checked with a 2100 Bioanalyzer (Agilent Technologies, Santa Clara, CA, USA). Operational taxonomic units (OTUs) were picked and clustered using the QIIME pipeline, and taxonomies were assigned based on the SILVA (v132) database at a 97% identity cutoff value [23]. OTU abundance information was normalized using a standard of sequence number corresponding to the sample with the fewest sequences. Downstream alpha (α) and beta (β) diversity analyses were performed based on this output-normalized data. Community alpha diversity indices were calculated using QIIME v1.9.1. UPGMA clustering was performed as a type of hierarchical clustering method to interpret the distance matrix using average linkage and was conducted using QIIME (v1.9.1).

### 2.3. Culture Media and Conditions

Carbohydrate-free semi-defined MRS [24] culture medium was used for the cultivation of *Bifidobacteria* and the fecal microbiome from samples. The medium composition was as follows (gL^−1^): bacteriological peptone (Sigma, Madrid, Spain) 10.0, yeast extract (ThermoFisher Scientific, Bleiswijk, The Netherlands) 5.0, sodium acetate (Sigma, Madrid, Spain) 5.0, ammonium citrate (VWR, Leuven, Belgium) 2.0, potassium phosphate (Merck, Darmstadt, Germany) 2.0, magnesium sulfate heptahydrate (VWR, Darmstadt, Germany) 0.366, manganese sulfate (Panreac, Barcelona, Spain) 0.05, and cysteine HCl (Acros Organics, Delphi, India) 2.5. The medium also included Tween 80 (Sigma, Madrid, Spain), 1 mL per liter. The medium was supplemented with 2% 2′-fucosyllactose. The pH was adjusted between 6.2 and 6.5, and the medium was autoclaved at 121 °C for 15 min. Selective and differential TOS-propionate (Merck, Darmstadt, Germany) medium was used to maintain and re-culture the bifidobacteria. All incubations were carried out at 37 °C in an anaerobic chamber (MG500, Don Whitley Scientific, West Yorkshire, UK, with an 80% *v*/*v* N_2_, 10% *v*/*v* CO_2_, and 10% *v*/*v* H_2_ atmosphere), unless otherwise specified.

### 2.4. 2′-Fucosyllactose Commercial Preparations

We used 2′-FL presented to us by Friesland Campina Ingredients (Paramus, NJ, USA). The 2′-FL was freshly diluted in MilliQ water at 30% (*w*/*v*) and sterilized by filtration through a pore size of 0.45 µm.

### 2.5. Screening of Co-Cultures from Different Fecal Microbiome Strains and B. bifidum in the Presence of Different Fibers and Food Extracts on PreBiome Plates^TM^

For the study, we used plates from the Biolog system. Biolog PreBiom^TM^ Technology offers a multidimensional phenotype profiling solution based on the impact of prebiotics on microbial function. The plates are 96-well microplates coated with various prebiotic substrates in triplicate. We used a PreBiomeM3 plate with dietary fibers and food extracts. Acell suspension from *B. bifidum*, co-culture (*B. bifidum*: fecal microbiome 1:1), and fecal microbiomes are dispensed into the PreBiome wells and incubated for 24 h. Cell growth was measured by measuring the optical density at 600 nm with a microplate reader, HIDEX, Finland.

### 2.6. Screening of Co-Cultures from Different Fecal Microbiome Strains and B. bifidum in the Presence of 2′-FL, Lactose, Fucose, Galactose, and Glucose

Co-cultures combining different fecal microbiomes and *Bifidobacterium bifidum* ATCC 29521 (final inoculum of 0.1% *v*/*v*) were carried out in semi-defined liquid MRS supplemented with 2′-FL, Lactose, Fucose, Galactose, and Glucose at a final concentration of 2% (*v*/*v*). The monocultures and co-cultures were performed in 96-well plates in triplicate. The growth kinetics were obtained using a Microplate Reader, HIDEX, Finland, by determining the OD 600 nm after 24 h.

### 2.7. Statistical Analysis and Software

Statistical analyses were conducted in R version 4.5.1 (R Core Team). A one-way analysis of variance (ANOVA) was performed to assess overall group differences. When significant effects were observed, Tukey’s Honest Significant Difference (HSD) test was applied for post hoc pairwise comparisons. Adjusted *p*-values were reported, with *p* ≤ 0.05 considered statistically significant; non-significant comparisons were labeled “ns.” Statistical analyses and visualization were carried out using the ggplot2, dplyr, tidyr, and ggsignif packages in R version 4.5.1. We also used the statistical program SPSS version 29 for analyzing the results of the cultivations.

## 3. Results

### 3.1. Bacterial Communities in the Studied Fecal Samples 

The bacterial sequences from 16S rRNA genes assigned to bacterial phyla and their relative abundance varied between the samples. After sequencing, we labeled the studied microbiomes as follows: A1- from infant 1 received a probiotic strain, B2- from infant 2, and C3-from the adult sample.

**Figure 1:**
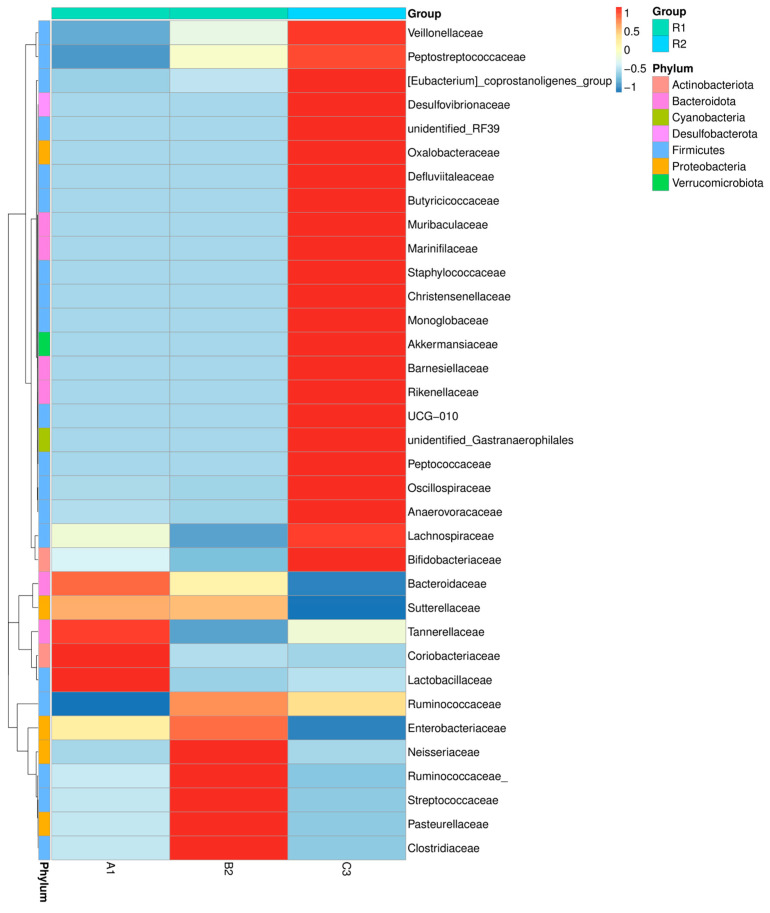
Taxa relative abundance at the family level in the studied fecal samples. *A1-from infant 1 received a probiotic strain*, *B2-from infant 2*, *and C3-from the adult sample*.

An increased presence of *Bifidobacteriaceae* families was reported in fecal sample (C3) compared to the other two fecal samples from infants (Figure 1). The *Lactobacillaceae* family is better represented in one of the two infant samples (A1). V3-V4 regions have been mostly used for identifying 16S rRNA gene sequences [25]. Highly represented in the adult sample are family members of the *Akkermansiaceae*. Previous studies have established in more detail the ability of *Akkermansia muciniphila* to digest specific oligosaccharides [26]. *Akkermansia muciniphila* is a widely researched anaerobic bacterium known for breaking down mucus and its positive links to human health. Previous studies have established that the bacterium can indeed grow on human milk and break down its HMOs. Proteomic analysis showed that when grown on human milk, expressed key enzymes—α-l-fucosidases, β-galactosidases, exo-α-sialidases, and β-acetylhexosaminidases—are capable of degrading HMO structures such as 2′-FL, LNT, lactose, and LNT2. This breakdown of host-derived glycans facilitates beneficial interactions with other bacteria in the gut, contributing to a supportive microbial network. Consequently, *A. muciniphila*’s ability to metabolize human milk likely aids its survival in the early-life intestine and helps it colonize the mucosal layer, thereby supporting long-term mucosal and metabolic health.

**Figure 2:**
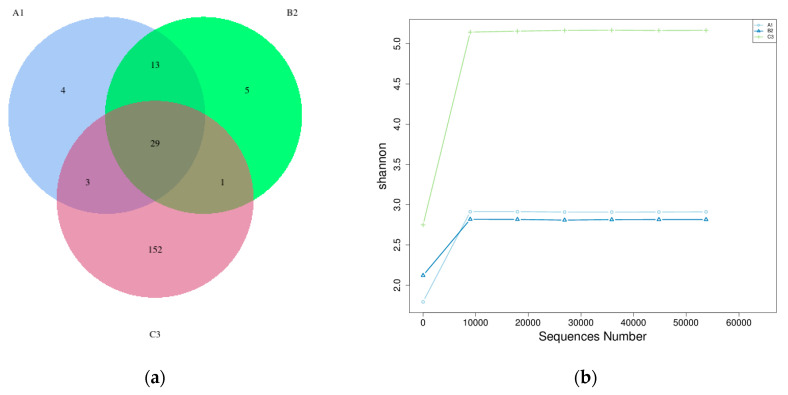
(**a**) Venn diagram showing compartmental core microbiota OTU distributions; (**b**) Rarefaction curves of sourdough samples and identified OTU numbers. *A1-from infant 1 received a probiotic strain*, *B2-from infant 2*, *and C3-from adult sample*.

In Figure 2, different colors in the diagram represent different groups. The number of overlapping parts between different color graphics is the number of OTUs shared between the two samples or two groups. Figure 2a shows 152 units represented in sample C3 and 13 units characteristic of the infant group. Microbial richness in the gut microbiome refers to the diversity of microorganisms present in the digestive system. A higher microbial richness score indicates a greater variety of bacteria, which can support digestion, nutrient absorption, and overall health—both physically and mentally. Scientists measure microbial richness using the Shannon Diversity Index (Figure 2b). A high richness score indicates a well-balanced gut microbiome with a variety of microorganisms. A diverse and stable microbial ecosystem is generally beneficial for health, aiding digestion, nutrient absorption, immune function, and defense against harmful pathogens. Conversely, a low richness score suggests a less diverse gut microbiome with fewer microbial species. This lack of diversity has been linked to health concerns, including obesity, autoimmune conditions, and gastrointestinal disorders.

**Figure 3:**
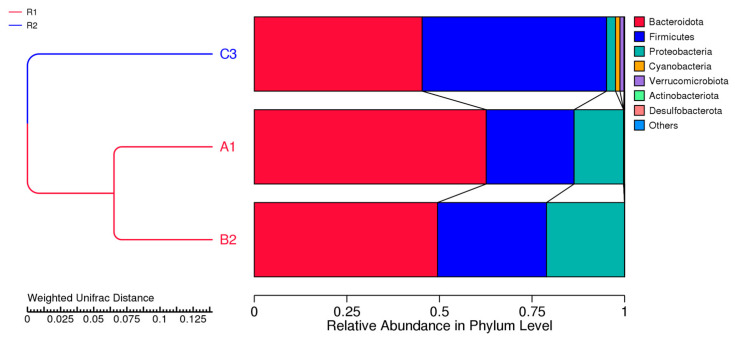
UPGMA cluster tree based on weighted UniFrac distance, along with species’ relative abundance and distribution at the phylum level. *A1-from infant 1 received a probiotic strain*, *B2-from infant 2*, *and C3-from adult sample*.

To study the similarity among different samples, clustering analysis was used to construct a cluster tree. The Unweighted Pair-group Method with Arithmetic Mean (UPGMA) is a hierarchical clustering method widely used in ecology for the classification of samples. A clustering analysis, combined with the creation of a clustering tree, was utilized to examine the similarities among the different samples. Figure 3 displays a UPGMA tree constructed using weighted UniFrac distances at the phylum level. On the left, you will see a hierarchical cluster tree, and on the right, each sample’s species abundance distribution is depicted. The findings indicate two primary clusters, one including A1 and B2, and another for C3, showing the highest similarity between samples (Figure 3). The samples A1 and B2 were primarily dominated by *Bacteroides*, but in sample C3, Firmicutes dominated. Sample C3 contains low levels of *Proteobacteria* compared to the other two samples.

### 3.2. Co-Cultivation and Influence of B.bifidum on the Absorption of Prebiotic Substrates

We studied the uptake and growth of individual microbiomes (fecal microbiome 1- from infant 1, fecal microbiome 2- from infant 2, and fecal microbiome 3 from the adult) when cultured in the presence of 28 different fibers and food extracts. We also separately cultured the *Bifidobacterium bifidum* strain on the same media. The results are presented in Table 1, Table 2 and Table 3. In the fecal microbiome from the adult sample, we found growth of almost all tested fibers and food extracts compared to the fecal microbiome from infants. In them, we did not observe changes in optical density values when grown on medium with added Oat Fiber Plus, Fibersol-2, Fiberpro 70L, GastroThera, Okra Extract Powder, White cabbage, Maize flour, Baobab, Zerose^®^ Erythritol Sweetener, and Quercetin.

When cultivating *Bifidobacterium bifidum*, we found an increase in the OD values in 8 of the tested substrates in the cultivation medium. When we conducted co-cultivation with the individual microbiomes and *Bifidobacteria*, we found better growth for some of the extracts and fibers. In a small proportion of cases, no statistical difference was reported with co-cultivation. 

**Figure 4:**
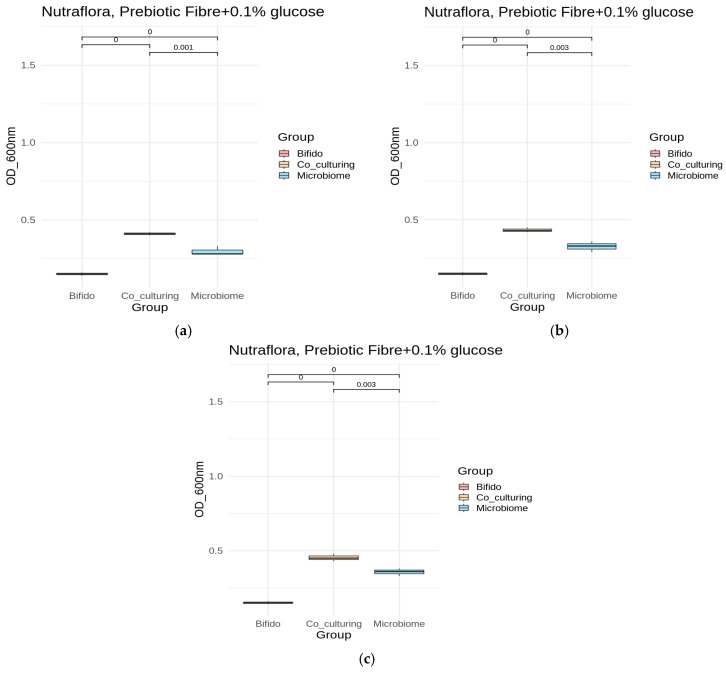
Cell growth: (**a**) microbiome A1, (**b**) microbiome B2, and (**c**) microbiome C3 in the presence of Nutriflora, prebiotic fiber + 0.15 glucose. Significant differences between mono- and co-cultures are indicated with different letters (*p*-value < 0.05).

Figure 4 presents the results of cell growth (OD 600 nm) in the presence of Nutriflora + 0.1% glucose, one of the prebiotic substrates used. Cultivation in its presence resulted in the most significant statistical differences between the growth of individual fecal microbiomes and the coculture with *B. bifidum*.

These results may form the basis for the production of new product combinations, including a specific prebiotic substrate. Functionally, the children’s microbiome is not yet in a state to absorb a significant portion of the fiber and food extracts introduced into their diet, but in combination with a specific probiotic strain, this process can be significantly facilitated.

**Figure 5:**
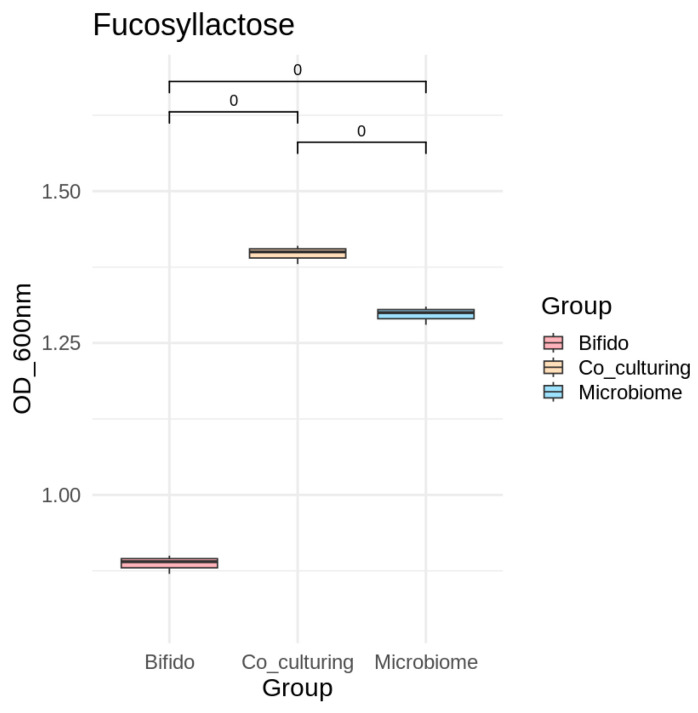
Cell growth by microbiome C3 in the presence of 2-FL. Significant differences between mono- and co-cultures are indicated with different letters (*p*-value < 0.05).

We analyzed the co-cultures of *B. bifidum* ATCC 29521 and three different fecal microbiomes in a liquid semi-defined MRS medium, supplemented with either 2′-fucosyllactose or its individual sugar components (lactose, fucose, galactose, and glucose). We then assessed microbial growth and carbohydrate consumption. After 24 h of incubation with 2′-FL, the co-cultures showed a significant increase in OD600 nm compared to Microbiome 1 and Microbiome 2 (*p*-value < 0.05, Table 2). However, when other carbon sources were used, microbial growth in co-cultures was similar to that observed in microbiomes (Table 4). These findings indicate a potential interaction between *B. bifidum* ATCC 29521 and the different strains represented in fecal microbiomes in infants in the presence of 2′-fucosyllactose. In all cultivations conducted in the presence of all sugars, we found statistical differences in the growth of mono- and coculture, Table 4 and Figure 5. 

## 4. Discussion

The impact of breastfeeding versus formula or mixed feeding on the gut microbiota is well documented. However, much less is known about how the microbiota shifts when solid foods begin to replace a milk-based diet [27]. A few longitudinal studies, each with repeated samples from a small group of infants, have shown that the microbial community undergoes significant changes during the period when solid foods are introduced and breastfeeding or formula feeding is phased out [28]. For example, in a study involving 330 Danish children from the ‘SKOT’ project, fecal samples collected at 9, 18, and 36 months revealed that the abundance of *Lactobacillaceae*, *Bifidobacteriaceae*, *Enterococcaceae*, and *Enterobacteriaceae* declined, while species from the *Lachnospiraceae*, *Ruminococcaceae*, and *Bacteroidaceae* families increased between 9 and 18 months—a time corresponding with the shift from milk to a family diet [29]. More recently, Thompson et al. observed that the introduction of solid foods was associated with increased gut microbial diversity in both exclusively breastfed and mixed-fed infants [30].

Over recent decades, there has been substantial debate over how exactly to define dietary fiber. A major point of contention has been whether to include oligosaccharides—carbohydrates made up of 3 to 9 monomeric units—that are resistant to digestion. According to the Nutrition Labeling Guidelines (revised in 2009), the term dietary fiber is officially defined as carbohydrate polymers made up of ten or more monomeric units, which cannot be broken down by the body’s own enzymes in the small intestine and are not absorbed there [31]. Dietary fibers are not digested in the upper parts of the gastrointestinal tract; instead, bacteria in the colon ferment them. Their fermentability and the specificity of the bacteria involved depend on various fiber characteristics—such as how many monomeric units they contain (i.e., degree of polymerization), how big the fiber particles are, whether they are soluble, their viscosity, among other properties [32]. Fiber consumption increases the diversity of bacteria that produce short-chain fatty acids (SCFAs), but different types of fibers support different microbes [33]. Studies have shown that using fibers such as inulin, guar gum, resistant starch, galacto-oligosaccharides (GOS), fructo-oligosaccharides (FOS), or arabinoxylan oligosaccharides leads to consistent growth in Bifidobacterium [34]. Other fiber types tend to encourage growth in Faecalibacterium, Ruminococcus (especially with resistant starch), Lactobacillus (notably when fibers include galactose or fructose units), Akkermansia, or Roseburia. These microbial shifts often appear within 1–2 weeks of the fiber intervention and remain stable for the duration of the study. Although in theory, boosting SCFA-producing bacteria by feeding them fiber in the colon should lead to higher SCFA levels in stool, many studies actually find the opposite. One explanation is that a large increase in stool volume (fecal bulk) from high fiber intake dilutes SCFA concentrations in feces [35,36].

A previous study examining the impact of 2′-fucosyllactose on the gut microbiota of infants identified distinct microbial profiles based on the microbiota’s ability to break down 2′-FL and the infant’s feeding method (breastfed or formula-fed). The study found that consuming 2′-FL significantly increased the presence of the *Lactobacillaceae* family, particularly in formula-fed infants’ fecal cultures [37]. Similar increases in *Lactobacilli* were reported by other researchers in infant fecal cultures containing 2′-FL. Additionally, an infant’s ability to metabolize various HMOs has been linked to a greater relative abundance of *Lactobacilli* in feces. However, the relationship between intestinal *Lactobacilli* and HMOs remains unclear, as *Lactobacilli*, unlike *Bifidobacteria*, have limited ability to utilize HMOs and hydrolyze terminal sugars [38]. Fucosyllactose notably shapes the infant gut microbiota by encouraging the growth of its main utilizers, *Bifidobacterium longum* ssp. *infantis* and *Bifidobacterium bifidum* [39]. Although to a lesser extent, strains such as *Bifidobacterium longum* subsp. *longum*, *Bifidobacterium dentium*, *Bifidobacterium kashiwanohense*, and *Bifidobacterium breve* also consume 2′FL. Additionally, other intestinal microorganisms lacking the specialized HMO uptake systems of bifidobacteria—such as *Bacteroides fragilis*, *Bacteroides vulgatus*, *Bacteroides thetaiotaomicron*, and *Akkermansia muciniphila*—can also break down 2′FL in pure culture, though their efficiency is lower, often leaving them at a competitive disadvantage compared to bifidobacteria [40]. Intestinal microbes that cannot directly use 2′FL can still break down its structural components, which are freed by the primary degraders along with some fermentation byproducts. This process promotes cross-feeding interactions in the infant gut. Much of this cross-feeding research has focused on the bifidobacterial community. For example, B. bifidum strains have been shown to release unconsumed 2′FL constituents in mono-culture, in HMO-supplemented fecal cultures, and in in vivo studies of the infant gut microbiome [41]. As a result, B. bifidum helps boost the growth of other Bifidobacterium species by releasing partially degraded HMO products, thereby increasing their prevalence in fecal communities. Similar outcomes were observed in vitro when four infant-derived *Bifidobacteria* (*B. bifidum R0071*, *B. breve M-16V*, *B. infantis R0033*, and *B. infantis M-63*) were grown with 2′FL [42]. The degradation of 2′-FL by bifidobacteria occurs through two different mechanisms: an intracellular, transport-dependent process in *B. longum* and an extracellular, glycosidase-dependent process in *B. bifidum* [39]. In the extracellular mechanism, glycosidase enzymes from *B. bifidum* release galactose and lactose into the surrounding environment, making these sugars accessible for lactobacilli growth. Several *B. bifidum* strains have been observed to accumulate degradation byproducts like galactose, fucose, or lactose when incubated with HMOs [43]. The exact process by which the intracellular breakdown of 2′-FL by bifidobacteria contributes to lactobacilli growth remains unclear. However, some studies have observed a temporary rise in monosaccharides and lactose levels in fermentation supernatants when *B. longum* or *B. infantis* metabolizes HMOs, despite the glycosidase activity occurring within the cells [7]. One possible explanation is that these internally generated saccharides are transported outside the cell to regulate osmotic pressure due to the rapid uptake of HMOs. Another possibility is the release of these sugars through bifidobacterial cell lysis. Nonetheless, the exact mechanisms of saccharide transport remain unknown [44].

A key factor in determining microbial dynamics in the microbiota is the sharing and/or competition for nutrients. In this context, microbe–microbe interactions can positively or negatively affect the fitness of the organisms present. In this study, we demonstrated that during growth on fucosyllactose, cross-feeding between multiple strains in different microbiomes and bifidobacteria increases the optical density at 600 nm, resulting from enhanced bacterial growth. In our future studies, it would be good to establish how the composition of the microbiome changes after cocultivation with a particular probiotic strain or combination of strains. Over time, the consumption of limiting nutrients and the release of acidic metabolites appear to shape the course of competition, thereby changing the relative abundance of species in the culture. 

## 5. Conclusions

Diet significantly influences the composition of the gut microbiome. The transition from breastfeeding to solid foods in the diet of infants also has a significant impact on the transforming microbiome. Since each person’s microbiome is different, it is important to study its functional characteristics and to study the effects of different diets on it. We used stool samples from infants and adults to investigate cross-species interactions contributing to microbiome-specific responses to one of the most abundant oligosaccharides in human milk. We found a significant difference in the absorption of 2-FL when co-cultivated with a probiotic strain in adults. We studied the effect of an additional 28 dietary substrates and fibers on the growth of strains in specific microbial consortia in the presence of a probiotic strain. When we conducted co-cultivation with the individual microbiomes and *Bifidobacteria*, we found better growth for some of the extracts and fibers. In a small proportion of cases, no statistical difference was reported with co-cultivation. One of the possible reasons for the better absorption of the indicated substrates and fibers may be cross-assimilation of absorption through various enzymes produced by the microbiome and supplemented by the probiotic strain.

Overall, the combined use of in vitro cultivation and genomic analyses can provide a clearer picture of the composition and changes in the composition of the infant microbiome, as well as changes in the functional characteristics of the microbiome. Further studies are needed to shed more light on how the presence of probiotics and prebiotic foods in co-cultivation would specifically influence changes in microbiome composition. 

## Figures and Tables

**Table 1:** Microbial growth (OD600 nm) of fecal microbiome 1 and co-cultivation in the presence of various fibers and food extracts.

Carbon Source	*B. bifidum*	Co-Culture	Fecal Microbiome 1
**Fibersol-2**	-	-	-
**Benefiber**	-	0.11 ± 0.09	0.22 ± 0.06
**Oat Fiber Plus**	-	-	-
**Apple (dietary fiber)**	-	0.16 ± 0.24	0.13 ± 0.10
**Full Fat Soya (dietary fiber)**	-	0.12 ± 0.25	0.21 ± 0.11
**Haricot beans (dietary fiber)**	-	0.18 ± 0.08	0.22 ± 0.27
**Fiberpro 70L**	-	-	-
**Nutriose^®^ FM 06**	-	0.31 ± 0.14	0.42 ± 0.05
**Fiberpro FOS 83**	0.10 ± 0.03	0.21 ± 0.13	0.19 ± 0.28
**Nutraflora, Prebiotic fiber**	0.13 ± 0.06	0.45 ± 0.19	0.40 ± 0.08
**Fiberpro FOS 83 + 0.1% glucose**	0.10 ± 0.07	0.31 ± 0.17	0.45 ± 0.41
**Nutraflora, Prebiotic fiber + 0.1% glucose**	0.15 ± 0.13	0.41 ± 0.21	0.29 ± 0.30
**GastroThera**	-	-	-
**Okra Extract Powder**	0.11 ± 0.27	0.11 ± 0.25	-
**PUREFRUIT Monk Fruit Extract**	0.11 ± 0.34	0.55 ± 0.12	0.48 ± 0.19
**Mussel tissue (trace elements)**	0.10 ± 0.09	0.30 ± 0.10	0.23 ± 0.37
**White cabbage (trace elements)**	-	-	-
**Maize flour (deoxynivalenol, blank)**	-	-	-
**PUREFRUIT Monk Fruit Extract + 0.1% glucose**	0.13 ± 0.04	0.54 ± 0.30	0.48 ± 0.11
**Mussel tissue (trace elements) + 0.1% glucose**	-	0.44 ± 0.26	0.48 ± 0.31
**Baobab**	-	-	-
**Brussels sprouts (vitamins)**	-	-	-
**Inavea BAOBAB ACACIA**	-	-	-
**Quercetin**	-	-	-
**Prebiotic Bifido Boost with Prebiotic Xylooligosaccharide (XOS) + 0.1% glucose**	-	0.29 ± 0.30	0.32 ± 0.11
**Beta-Sitosterol, Phytosterols capsules**	-	-	-
**Prebiotic Bifido Boost with PreticX Xylooligosaccharide (XOS)**	-	0.51 ± 0.32	0.49 ± 0.07
**Zerose^®^ Erythritol Sweetener**	-	-	-

**Table 2:** Microbial growth (OD600 nm) of fecal microbiome 2 and co-cultivation in the presence of various fibers and food extracts.

Carbon Source	*B. bifidum*	Co-Culture	Fecal Microbiome 2
**Fibersol-2**	-	-	-
**Benefiber**	-	-	-
**Oat Fiber Plus**	-	-	-
**Apple (dietary fiber)**	-	0.28 ± 0.21	0.23 ± 0.10
**Full Fat Soya (dietary fiber)**	-	0.14 ± 0.13	0.12 ± 0.19
**Haricot beans (dietary fiber)**	-	0.14 ± 0.08	0.14 ± 0.21
**Fiberpro 70L**	-	-	-
**Nutriose^®^ FM 06**	-	0.21 ± 0.09	0.18 ± 0.01
**Fiberpro FOS 83**	0.10 ± 0.03	0.20 ± 0.14	0.19 ± 0.31
**Nutraflora, Prebiotic fiber**	0.13 ± 0.06	0.28 ± 0.15	0.40 ± 0.22
**Fiberpro FOS 83 + 0.1% glucose**	0.10 ± 0.07	0.29 ± 0.12	0.14 ± 0.51
**Nutraflora, Prebiotic fiber + 0.1% glucose**	0.15 ± 0.13	0.43 ± 0.24	0.33 ± 0.31
**GastroThera**	-	-	-
**Okra Extract Powder**	0.11 ± 0.27	0.11 ± 0.25	-
**PUREFRUIT Monk Fruit Extract**	0.11 ± 0.34	0.32 ± 0.13	0.47 ± 0.10
**Mussel tissue (trace elements)**	0.10 ± 0.09	0.35 ± 0.09	0.24 ± 0.07
**White cabbage (trace elements)**	-	-	-
**Maize flour (deoxynivalenol, blank)**	-	-	-
**PUREFRUIT Monk Fruit Extract + 0.1% glucose**	0.13 ± 0.04	0.57 ± 0.31	0.51 ± 0.12
**Mussel tissue (trace elements) + 0.1% glucose**	-	0.43 ± 0.20	0.52 ± 0.43
**Baobab**	-	-	-
**Brussels sprouts (vitamins)**	-	0.21 ± 0.09	0.21 ± 0.07
**Inavea BAOBAB ACACIA**	-	-	-
**Quercetin**	-	-	-
**Prebiotic Bifido Boost with PrebioticX Xylooligosaccharide (XOS) + 0.1% glucose**	-	0.37 ± 0.34	0.42 ± 0.10
**Beta-Sitosterol, Phytosterols capsules**	-	-	-
**Prebiotic Bifido Boost with PreticX Xylooligosaccharide (XOS)**	-	0.52 ± 0.33	0.38 ± 0.07
**Zerose^®^ Erythritol Sweetener**	-	-	-

**Table 3:** Microbial growth (OD600 nm) of fecal microbiome 3 and co-cultivation in the presence of various fibers and food extracts.

Carbon Source	*B. bifidum*	Co-Culture	Fecal Microbiome 3
**Fibersol-2**	-	0.14 ± 0.12	0.12 ± 0.08
**Benefiber**	-	0.14 ± 0.11	0.13 ± 0.07
**Oat Fiber Plus**	-	-	-
**Apple (dietary fiber)**	-	0.18 ± 0.24	0.12 ± 0.14
**Full Fat Soya (dietary fiber)**	-	0.17 ± 0.12	0.13 ± 0.16
**Haricots beans (dietary fiber)**	-	0.15 ± 0.14	0.13 ± 0.24
**Fiberpro 70L**	-	0.13 ± 0.05	0.11 ± 0.01
**Nutriose^®^ FM 06**	-	0.20 ± 0.07	0.14 ± 0.07
**Fiberpro FOS 83**	0.10 ± 0.03	0.24 ± 0.10	0.28 ± 0.32
**Nutraflora, Prebiotic fiber**	0.13 ± 0.06	0.28 ± 0.12	0.39 ± 0.21
**Fiberpro FOS 83 + 0.1% glucose**	0.10 ± 0.07	0.31 ± 0.08	0.47 ± 0.25
**Nutraflora, Prebiotic fiber + 0.1% glucose**	0.15 ± 0.13	0.45 ± 0.25	0.36 ± 0.34
**GastroThera**	-	0.23 ± 0.24	0.22 ± 0.27
**Okra Extract Powder**	0.11 ± 0.27	0.45 ± 0.27	0.40 ± 0.45
**PUREFRUIT Monk Fruit Extract**	0.11 ± 0.34	0.57 ± 0.32	0.49 ± 0.09
**Mussel tissue (trace elements)**	0.10 ± 0.09	0.36 ± 0.08	0.29 ± 0.07
**White cabbage (trace elements)**	-	0.33 ± 0.22	0.31 ± 0.06
**Maize flour (deoxynivalenol, blank)**	-	0.21 ± 0.51	0.15 ± 0.02
**PUREFRUIT Monk Fruit Extract + 0.1% glucose**	0.13 ± 0.04	0.61 ± 0.34	0.52 ± 0.14
**Mussel tissue (trace elements) + 0.1% glucose**	-	0.46 ± 0.21	0.52 ± 0.41
**Baobab**	-	0.40 ± 0.02	0.32 ± 0.03
**Brussels sprouts (vitamins)**	-	0.28 ± 0.09	0.28 ± 0.04
**inavea BAOBAB ACACIA**	-	0.13 ± 0.06	0.11 ± 0.24
**Quercetin**	-	0.13 ± 0.12	0.12 ± 0.34
**Prebiotic Bifido Boost with PrebioticX Xylooligosaccharide (XOS) + 0.1% glucose**	-	0.49 ± 0.34	0.43 ± 0.08
**Beta-Sitosterol, Phytosterols capsules**	-	0.15 ± 0.32	0.13 ± 0.06
**Prebiotic Bifido Boost with PreticX Xylooligosaccharide (XOS)**	-	0.47 ± 0.36	0.34 ± 0.04
**Zerose^®^ Erythritol Sweetener**	-	0.13 ± 0.21	0.1 ± 0.08

**Table 4:** Microbial growth (OD600 nm) of mono-cultures and co-cultures of B. bifidum ATCC 29521 and three different fecal microbiomes in MRS supplemented with carbohydrate constituent of 2-FL after 24 h of incubation. Significant differences between mono- and co-cultures are indicated with different letters (*p*-value < 0.05).

Carbon Source	*B. bifidum* ATCC 29521	Co-Culture *B. bifidum* + Microbiome A1	Fecal Microbiome A1
**2′-fucosyllactose**	0.89 ± 0.12	1.3 ± 0.14	1.1 ± 0.09
**Fucose**	0.30 ± 0.15	0.52 ± 0.06	0.63 ± 0.08
**Galactose**	0.43 ± 0.08	1.17 ± 0.07	1.29 ± 0.09
**Glucose**	1.12 ± 0.09	1.49 ±0.1	1.52 ± 0.05
**Lactose**	1.15 ± 0.13	1.16 ±0.03	1.20 ± 0.04
**Carbon Source**	***B. bifidum*** **ATCC 29521**	**Co-Culture** ***B. bifidum*** **+ microbiome B2**	**Fecal microbiome B2**
**2′-fucosyllactose**	0.89 ± 0.12	1.4 ± 0.05	1.3 ± 0.09
**Fucose**	0.29 ± 0.15	0.80 ± 0.04	0.85 ± 0.08
**Galactose**	0.45 ± 0.08	1.15 ± 0.02	1.1 ± 0.03
**Glucose**	1.12 ± 0.09	1.3 ± 0.04	1.4 ± 0.1
**Lactose**	1.15 ± 0.13	1.1 ± 0.04	1.3 ± 0.03
**Carbon Source**	***B. bifidum*** **ATCC 29521**	**Co-Culture** ***B. bifidum*** **+ microbiome C3**	**Fecal microbiome C3**
**2′-fucosyllactose**	0.89 ± 0.12	1.3 ± 0.11	0.9 ± 0.09
**Fucose**	0.29 ± 0.15	0.87 ± 0.08	0.74 ± 0.03
**Galactose**	0.45 ± 0.08	1.2 ± 0.01	1.5 ± 0.0
**Glucose**	1.12 ± 0.09	1.58 ± 0.13	1.6 ± 0.09
**Lactose**	1.15 ± 0.13	1.20 ± 0.12	1.32 ± 0.07

## Data Availability

The original contributions presented in this study are included in the article. Further inquiries can be directed towards the corresponding author.

## Abbreviations

The following abbreviations are used in this manuscript:

HMOs	Human milk oligosaccharides
GOS	Galactooligosaccharides
SCFAs	Short-chain fatty acids
FF	Formula-fed
FOS	Fructooligosaccharides
2-FL	2′-fucosyllactose
